# Colloidal Fouling of Nanofiltration Membranes: Development of a Standard Operating Procedure

**DOI:** 10.3390/membranes7010004

**Published:** 2017-01-18

**Authors:** Md Abdullaha Al Mamun, Subir Bhattacharjee, David Pernitsky, Mohtada Sadrzadeh

**Affiliations:** 1 Department of Mechanical Engineering, 6-074 NINT Building, University of Alberta, Edmonton, AB T6G 2G8, Canada; mdabdull@ualberta.ca; 2 Water Planet Engineering, 721 S Glasgow Ave, Inglewood, CA 90301, USA; subir@waterplanet.com; 3 Suncor Energy Inc., P.O. Box 2844, 150-6th Ave. SW, Calgary, AB T2P 3E3, Canada; dpernitsky@suncor.com

**Keywords:** nanofiltration, membrane, colloidal fouling, water treatment, osmotic pressure, critical flux

## Abstract

Fouling of nanofiltration (NF) membranes is the most significant obstacle to the development of a sustainable and energy-efficient NF process. Colloidal fouling and performance decline in NF processes is complex due to the combination of cake formation and salt concentration polarization effects, which are influenced by the properties of the colloids and the membrane, the operating conditions of the test, and the solution chemistry. Although numerous studies have been conducted to investigate the influence of these parameters on the performance of the NF process, the importance of membrane preconditioning (e.g., compaction and equilibrating with salt water), as well as the determination of key parameters (e.g., critical flux and trans-membrane osmotic pressure) before the fouling experiment have not been reported in detail. The aim of this paper is to present a standard experimental and data analysis protocol for NF colloidal fouling experiments. The developed methodology covers preparation and characterization of water samples and colloidal particles, pre-test membrane compaction and critical flux determination, measurement of experimental data during the fouling test, and the analysis of that data to determine the relative importance of various fouling mechanisms. The standard protocol is illustrated with data from a series of flat sheet, bench-scale experiments.

## 1. Introduction

Separation of dissolved and suspended matter from a solvent constitutes a major unit operation, and is important in virtually every industry, including water treatment, environmental remediation, resource extraction, food processing, and effluent treatment. Membrane based separation processes have become extremely popular owing primarily to their lower operating expenses and lower energy consumption compared to other processes, such as distillation. Other advantages of membrane processes include (1) easy integration with other types of separation processes; (2) availability of a variety of membrane materials with different properties that enables tailored separation for targeted components; (3) high rejection of dissolved solutes and ions; and (4) compact design.

Among different membrane processes, pressure driven filtration processes, classified as microfiltration (MF), ultrafiltration (UF), nanofiltration (NF), and reverse osmosis (RO), are widely used for separating constituents from the liquid phase. MF and UF processes are used for separation of particles and macromolecules via physical retention (sieving) of the suspended matter by microporous membranes based on particle size. Typically, MF membranes retain particles >100 nm, and have large pore sizes, whereas UF membranes retain macromolecules, colloids, and proteins in size range of 5–100 nm. These two processes are not suitable for rejection of salts and divalent ions, as the pore size of the membranes used is larger than these entities. RO has been widely used in desalination of water for the past four decades due to its high rejection of monovalent salts [1] which are removed via a solution-diffusion mechanism. Because of higher hydraulic resistance and osmotic pressure development, RO plants must operate at very high pressures, making the process energy intensive.

NF processes act as a bridge between UF and RO. NF membranes provide a higher permeability and a lower rejection of monovalent ions (<70%) compared to RO processes, but offer a reasonable rejection of multivalent ions (>99%) and organic matter (>90%) [2,3]. NF membranes are generally classified according to the size of contaminants they remove. “Tight” NF membranes can approach the salt rejection of RO membranes, whereas “loose” NF membranes can be similar in performance to UF membranes [4]. NF processes provide a facile method for softening, and even for partial desalination of brackish waters, while employing considerably lower operating pressures than RO.

The major challenge of the pressure driven membrane processes is reduced separation performance due to fouling. Fouling may be defined as the irreversible deposition of retained particles, colloids, macromolecules, salt, etc. on the surface or within the pores of membranes [5]. This includes adsorption [6,7,8], pore blocking [9,10], precipitation or scaling [11,12] and cake formation [13,14]. Fouling mechanisms during MF and UF processes typically include pore blocking, solute adsorption, and cake/gel formation. In the salt rejecting NF and RO membranes, however, fouling is mainly governed by the adsorption of contaminants on the surface and scaling by divalent ions like Ca^2+^ and Mg^2+^. Fouling reduces membrane performance and longevity as well as flux and permeate quality, and subsequently increases operating costs [3].

A particular problem of interest in the context of NF process is the combined fouling due to cake formation by charged colloids retained by the membrane, and concentration polarization (CP) due to the retained ions. Such fouling mechanisms are evident in numerous NF processes like desalination, water treatment, softening, produced water treatment in petroleum extraction and refining, etc. [14,15,16]. The cake formation and the CP phenomena are not additive, but manifest in a more complex manner, depending on the particle charge, particle size, ion concentration in the feed, membrane characteristics, and the influence of operating and hydrodynamic conditions [17,18,19,20,21].

Although many studies have been conducted to provide insight into the effect of physicochemical parameters that synergistically influence colloidal fouling, the importance and methodology of membrane preconditioning (e.g., membrane compaction) and the determination of substantial parameters (e.g., critical flux) before fouling experiment have not been reported. The aim of this paper is to present a standard experimental and data analysis protocol for NF colloidal fouling experiments. The developed methodology covers preparation and characterization of water samples and colloidal particles, pre-test membrane compaction and critical flux determination, measurement of experimental data during the fouling test, and the analysis of that data to determine the relative importance of various fouling mechanisms. The standard protocol is illustrated with data from a series of flat sheet, bench-scale experiments.

## 2. Theoretical Background and Development of Data Analysis Model

In salt rejecting NF/RO membranes, the CP by salt ions and fouling by colloidal particles, organic matter, and microorganisms are two interconnected phenomena that reduce the water flux through the membranes. Hoek and Elimelech have postulated the first model, namely, cake enhanced concentration polarization (CECP), to elucidate the mechanism of flux decline by the combined effect of CP and colloidal fouling [14]. They explained the fouling mechanism as arising from hindered back-diffusion of salt ions within the colloidal deposit layers, resulting in an increase of CP as well as the transmembrane osmotic pressure (TMOP). In this section, the engineering basis and the mathematical equations used in the data analysis for the quantification of fouling based on CECP model are presented. These equations are used in the subsequent sections to analyze experimental data from a series of bench-scale experiments to determine the fouling mechanisms present. The NF experiments were conducted using 65 nm radius silica particles with 2300 kg/m^3^ density and ~−30 mV surface charge (at pH 7.0 in 10 mM NaCl solution) and NF membranes with the effective surface area of 140 cm^2^ and ~18 mV surface charge (at pH 7.0 in 10 mM NaCl solution).

Resistance through a NF membrane is made up of three major components: the hydrodynamic resistance of the membrane in the absence of foulants, the resistance due to the accumulation of ions at the membrane surface (concentration polarization), and the resistance due to the accumulation of colloids at the membrane surface (cake layer fouling).

*Membrane Resistance*: The hydrodynamic resistance of the membrane itself in the absence of foulants is determined by measuring flow and pressure over time through the membrane using pure water, and calculating the pure water flux ($\nu_{w}^{0}$, m^3^/m^2^s). Membrane resistance (*R*_m_, 1/m) is then calculated using the following equation:

(1) $$\nu_{w}^{0}=\frac{\Delta P}{\mu R_{m}}$$

where *μ* is the dynamic viscosity of the pure water (Pa·s) and Δ*P* is the transmembrane pressure (Pa).

*Concentration Polarization*: The resistance due to the accumulation of ions at the membrane surface (CP), in the absence of colloids, is captured by the generated TMOP. The TMOP reduces the effective pressure driving force for solvent transport and is determined by measuring flow, pressure, and permeate salt concentration over time through the membrane using a salt water solution with a specific NaCl concentration. From this experimental data, the salt water flux ($\nu_{w}^{s}$, m^3^/m^2^s) and the observed salt rejection (*R*_o_) can be calculated. The resistance due to the effects of the accumulated ions, the TMOP (Δ*π*, Pa), can then be calculated using the following equations:

(2) $$R_{o}=1-\frac{C_{i,p}}{C_{i,f}}$$

(3) $$\nu_{w}^{s}=\frac{\Delta P-\Delta \pi }{\mu R_{m}}$$

where *C*_i,f_ and *C*_i,p_ (mol/m^3^) are the salt concentration in the feed and permeate, respectively. Using evaluated *R*_o_ and Δ*π*, the initial electrolyte mass transfer coefficient in the salt CP layer (*k*_i_, m/s) is then calculated combining the van’t Hoff equation and film theory (see Appendix A for the derivation of this equation):

(4) $$\Delta \pi =2RTC_{i,f}R_{o}\exp\left(\nu_{w}^{s}/k_{i}\right)$$

where *R* is the universal gas constant (J/mol K) and *T* is the absolute temperature of water (K).

*Cake Layer Fouling*: The cake layer hydrodynamic resistance (*R*_c_, 1/m) due to the accumulation of colloidal particles at the membrane surface can be evaluated by calculating the mass of colloids deposited on the membrane (*M*_c_, kg) using a simple mass balance around the membrane and then estimating the hydrodynamic drag exerted by that mass of spherical colloids within the cake layer. The Kuwabara cell model [22] can be used to estimate the hydraulic resistance through the cake layer:

(5) $$R_{c}=\frac{9A_{K}M_{c}}{2a^{2}g^{*}\rho_{p}A_{m}}=\frac{9A_{K}\delta_{c}(1-\varepsilon_{c})}{2a^{2}g^{*}}$$

where *ε*_c_ is the average cake layer porosity, $\delta_{c}$ is the thickness of cake layer (m), *a* is the particle diameter (m), *A*_m_ is the effective membrane area (m^2^), *A*_K_ is the Kuwabara correction factor accounting for the effect of neighboring particles in the cake layer, and g* accounts for electroosmotic effect in swarm of charged colloidal particles [23]. The parameter g* quantifies this effect in terms of an electroviscous resistance, which is additional to the hydrodynamic resistance of the cake layer. It also directly relates the cake volume fraction ($\phi_{c}$) and zeta potential of particles (*ψ*_p_) to the electroviscous resistance. Our previous studies showed that the electroosmotic effect becomes significant for cake layers with higher cake volume fraction and zeta potential [23]. For $\phi_{c}$ ~ 0.5 and *ψ*_p_ ~ −30 mV in the present study, there would be no electroosmotic backflow and thus g* = 1 [23]. The expressions of *A*_K_ and *M*_c_ are given as follows:

(6) $$A_{K}=\frac{1}{1-\frac{9}{5}\phi_{c}^{1/3}+\phi_{c}-\frac{1}{5}\phi_{c}^{2}}$$

(7) $$M_{c}=\rho_{p}\phi_{c}\delta_{c}A_{m}$$

In these equations, $\phi_{c}$ is the cake volume fraction ($\phi_{c}=1-\varepsilon_{c}$), and $\rho_{p}$ is the density of colloidal particles (2300 kg/m^3^ in this study).

*Cake Enhanced Osmotic Pressure*: Knowing the membrane resistance from the pure water flux experiment and the TMOP due to concentration polarization from the salt water flux experiment, the overall cake enhanced osmotic pressure (CEOP) resulting from the combined effects of the retained colloids and the retained ions can be determined from the actual membrane experimental data. According to film theory, the permeate flux in the fouling experiment can be written as:

(8) $$\nu_{w}=\frac{\Delta P_{c}}{\mu \;R_{c}}=\frac{\Delta P_{m}}{\mu \;R_{m}}=\frac{\Delta P_{t}-\Delta \pi_{m}}{\mu \;\left(R_{m}+R_{c}\right)}$$

where $\Delta P_{t}$, $\Delta P_{c}$, and $\Delta P_{m}$ (Pa) are the total, trans-cake, and trans-membrane hydraulic pressures, respectively. The total applied pressure ($\Delta P_{t}$ as the driving force of transport through the membrane) is the summation of the trans-cake hydraulic pressure ($\Delta P_{c}$), the trans-membrane pressure ($\Delta P_{m}$), and CEOP ($\Delta \pi_{m}$). By knowing the membrane resistance (*R*_m_), cake resistance (*R*_c_), and permeate flux, the CEOP is calculated as follows:

(9) $$\Delta \pi_{m}=\Delta P-\nu_{w}\mu \left(R_{m}+R_{c}\right)$$

The CEOP can also be calculated based on the modified van’t Hoff equation,

(10) $$\Delta \pi_{m}=2RTC_{i,f}R_{i,o}\exp\left(\nu_{w}/k_{i}^{*}\right)$$

where $k_{i}^{*}$ (m/s) is the hindered mass transfer coefficient ($k_{i}^{*}$) which consists of two parts: one describes the mass transfer within the cake layer (*δ*_c_), which can be considered as internal mass transfer coefficient, and the other one is related to the mass transfer within the CP layer (between the surface of cake layer and the bulk, *δ*_s_ = *δ* − *δ*_c_), which is considered as external mass transfer coefficient [14,17,24]:

(11) $$\frac{1}{k_{i}^{*}}=\frac{\delta_{c}}{D_{i}^{*}}+\frac{\delta_{s}}{D_{i}}=\frac{\delta_{c}}{D_{i}^{*}}-\frac{\delta_{c}}{D_{i}}+\frac{\delta }{D_{i}}$$

where *δ* (m) is the thickness of the mass boundary layer, $D_{i}$ (m^2^/s) is the bulk diffusivity, *δ*/*D*_i_ is the inverse of mass transfer coefficient within the CP layer (1/*k*_i_), and $D_{i}^{*}$ (m^2^/s) is the hindered diffusivity. Hence, the following equation is derived for the hindered mass transfer coefficient:

(12) $$\frac{1}{k_{i}^{*}}=\left[\delta_{c}\left(\frac{1}{D_{i}^{*}}-\frac{1}{D_{i}}\right)+\frac{1}{k_{i}}\right]$$

The hindered diffusivity ($D_{i}^{*}$) in Equation (12) is related to bulk diffusivity ($D_{i}$), tortuosity (*ζ*), and cake porosity ($\varepsilon_{c}$) as $D_{i}^{*}=D_{i}\varepsilon_{c}\varsigma^{-1}$. Tortuosity in the present work is also related to porosity by $\varsigma =1-\ln\varepsilon_{c}^{2}$ [17,24]. Hence, Equations (9) and (10) are both related to cake porosity (or cake volume fraction) which is calculated by setting these two equations equal [23]. After finding the cake porosity, CEOP is calculated using either of these equations.

The pressure drops are non-dimensionalized by dividing them by the applied transmembrane pressure ($\Delta P_{t}$). Hence, the summation of non-dimensional trans-cake pressure ($\Delta P_{c}^{*}$), trans-membrane pressure ($\Delta P_{m}^{*}$), and CEOP ($\Delta \pi_{m}^{*}$) can be expressed as follows:

(13) $$\Delta P_{c}^{*}+\Delta P_{m}^{*}+\Delta \pi_{m}^{*}=1$$

## 3. Results and Discussion

### 3.1. Size and Zeta Potential of Silica Particles

**Figure 1:**
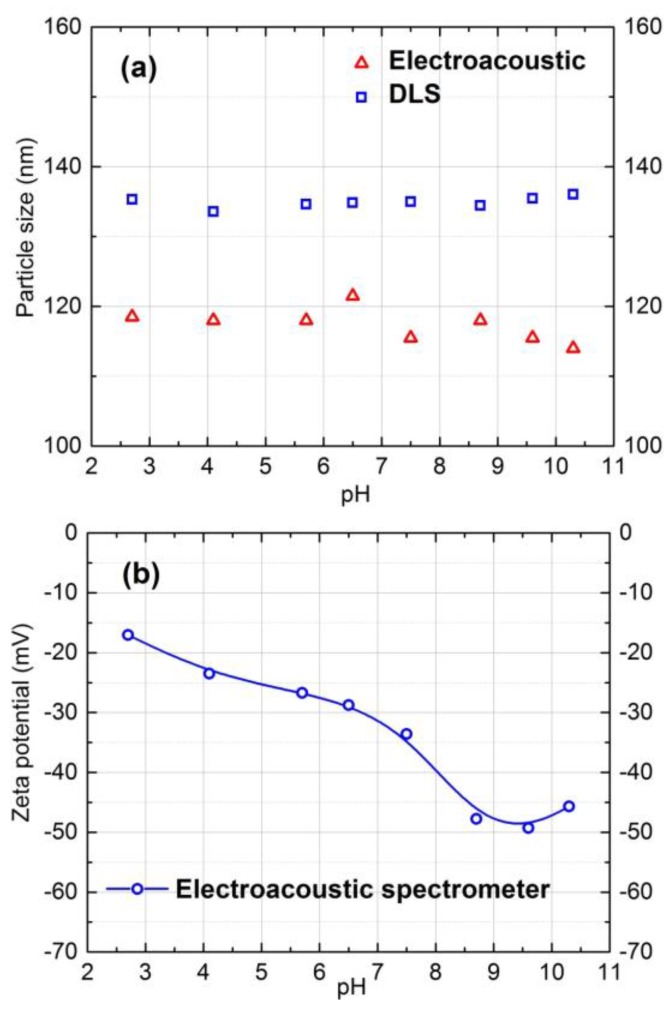
Particle size and zeta potential of model silica colloidal particles as a function of pH in 10 mM NaCl solution at 25 °C.

The size and zeta potential of the silica particles were measured and plotted as a function of pH in Figure 1. The DLS measurement in Figure 1a provides the hydrodynamic diameter of the particles. The size of particles varied from 115 nm to 145 within the pH range of 2.0–10.5 in 10 mM NaCl concentration. Therefore the utilized silica particles in this study were stable for this pH range in 10 mM NaCl ionic strength solution.

According to Figure 1b, negative zeta potential of silica particles increased with increasing pH. The zeta potential values within the pH 7.0 to 9.0 in 10 mM NaCl solution varied from −30 to −45 mV. The same characterization procedure was repeated for 20 mM salt solution, and almost the same results were obtained.

### 3.2. Membrane Compaction Results

The swelling and compaction tendency of the polymeric membrane matrix determines the pure water permeability at different pressures. The intrusion of water molecules into the polymer swells the polymer matrix and increases water flux by increasing the diffusion rates at higher pressures. In contrast, membrane compaction under an applied pressure decreases the fractional free volume within the polymer matrix and leads to a denser structure. As a consequence, the water flux decreases. The combined effect of swelling and compaction determines the permeability and hydraulic resistance of a polymeric membrane.

**Figure 2:**
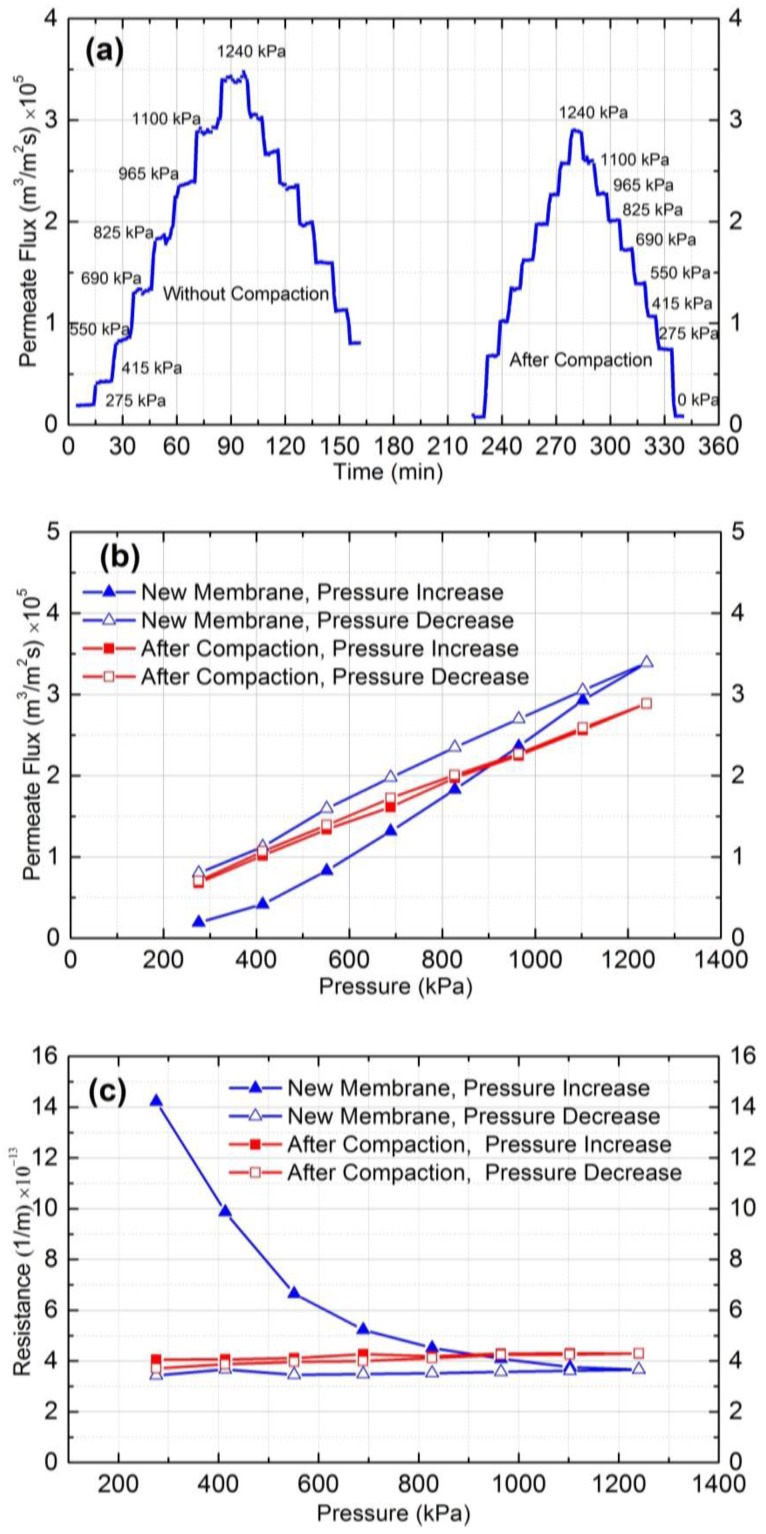
(**a**) Permeate flux vs. time at different pressure before and after compaction; (**b**) permeate flux vs. pressure (kPa); and (**c**) resistance (1/m) vs. pressure (kPa).

Figure 2a shows the permeate flux results of a new and a used NF90 membrane over time at different operating pressures. As can be seen, for a new membrane, different flux values were obtained at the same pressures in the upward and downward measurements. However, symmetric permeate flux versus time plot was obtained for the compacted membrane, showing small hysteresis and stable values at different pressures.

To further clarify the compaction effect, the permeate flux and resistance of the new and the compacted membranes were plotted against the applied pressure as shown in Figure 2b,c, respectively. According to these figures, the permeate flux increased, and hydraulic resistance decreased by increasing the applied pressure for the new membrane. The decline in the rate of resistance at higher pressure was small, and it became almost constant at 1100 and 1240 kPa. This behavior can be attributed to the dual effect of membrane swelling and compaction on permeate flux by increasing the applied pressure. Increasing the pressure increases both compaction and swelling of the membrane. In the case of a new membrane, the effect of swelling is dominant and results in a non-linear flux vs. pressure graph (Figure 2b), as well as a decreasing trend for hydraulic resistance with increasing pressure (Figure 2c). At higher pressures, the counter effects of swelling and compaction are equal, which leads to constant hydraulic resistance. Hysteresis can also be observed in Figure 2b,c. For the compacted membrane, the permeate flux changed linearly, and resistance remained constant both for the increasing and decreasing trends of pressure, which shows no hysteresis after compaction at higher pressure. The average hydraulic resistance of the compacted membranes was 4.0 ± 0.1 × 10^13^ m^−1^. Therefore, membrane compaction at a pressure greater than fouling experiments pressure (which was estimated by critical flux experiments) must be conducted before each experiment.

In summary, in order to ensure that experimental data is not influenced by compaction during the fouling test, it is essential that membrane compaction must be conducted before each fouling experiment. Compaction must be conducted at a pressure greater than the pressure intended to be used for the fouling experiments. The proper pressure must also be set based on achieving the critical flux, as will be discussed in the next section.

### 3.3. Critical Flux Measurement

**Figure 3:**
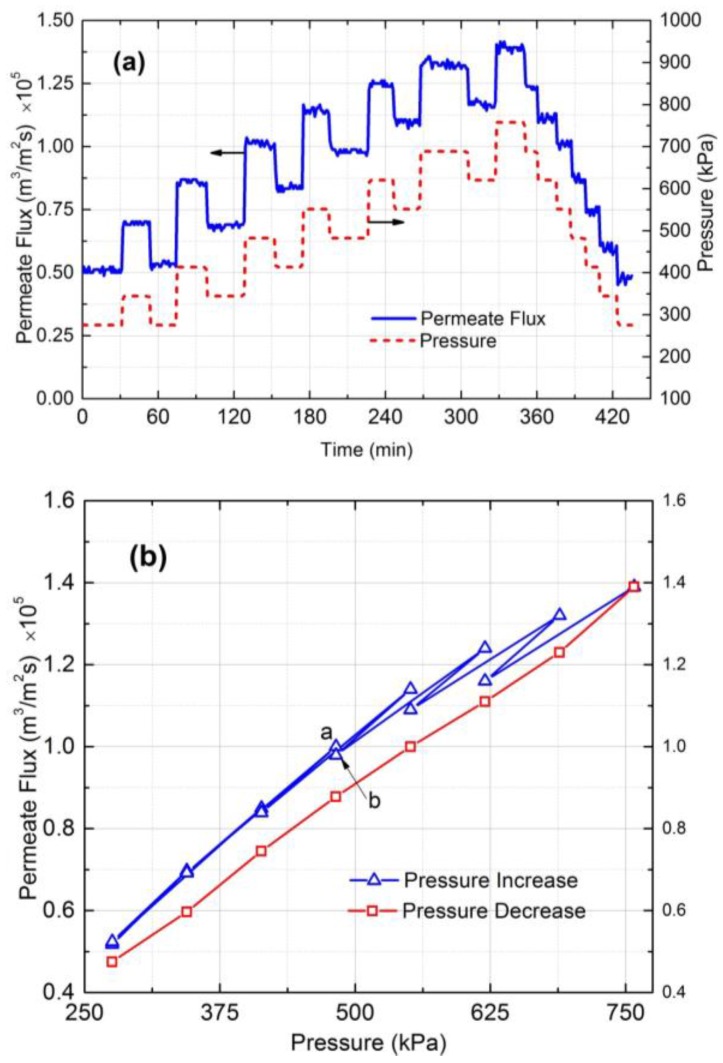
(**a**) Pressure and permeate flux vs. time and (**b**) permeate flux vs. pressure for 130 nm silica particles in 10 mM NaCl solution. The cross-flow velocity was 0.1 m/s and Reynolds number was 344.

To find the critical flux the applied pressure and permeate flux were plotted against time (Figure 3a). The permeate flux vs. pressure graph was also plotted (Figure 3b). The goal is to find a point where irreversible fouling or cake formation occurs. The experimental results shows that the permeate flux was reversible at pressures up to 482 kPa. The irreversible fouling or cake layer formation first appeared at 550 kPa, and at 620 kPa the irreversibility became significant. Therefore, based on Figure 3b, the critical flux for irreversible cake formation is around 1.16 × 10^−5^ m^3^/m^2^s.

### 3.4. Fouling Experiment Results

The application of the data analysis model developed earlier is illustrated below using the experimental results from five bench-scale NF experiments. These experiments were designed to study the effects of transmembrane pressure, salt concentration, cross-flow velocity, and colloidal particle concentration on membrane fouling behaviour. The transient flux decline was determined for each experiment over a duration of 5 h. Simultaneously, the deposited mass of the cake layer, as well as the observed salt rejection were recorded as a function of time. All experiments were conducted at the same pH and temperature, which maintained the particle size and zeta potential fixed. The experimental condition is presented in Table 1.

#### 3.4.1. Effect of Silica Concentration

**Figure 4:**
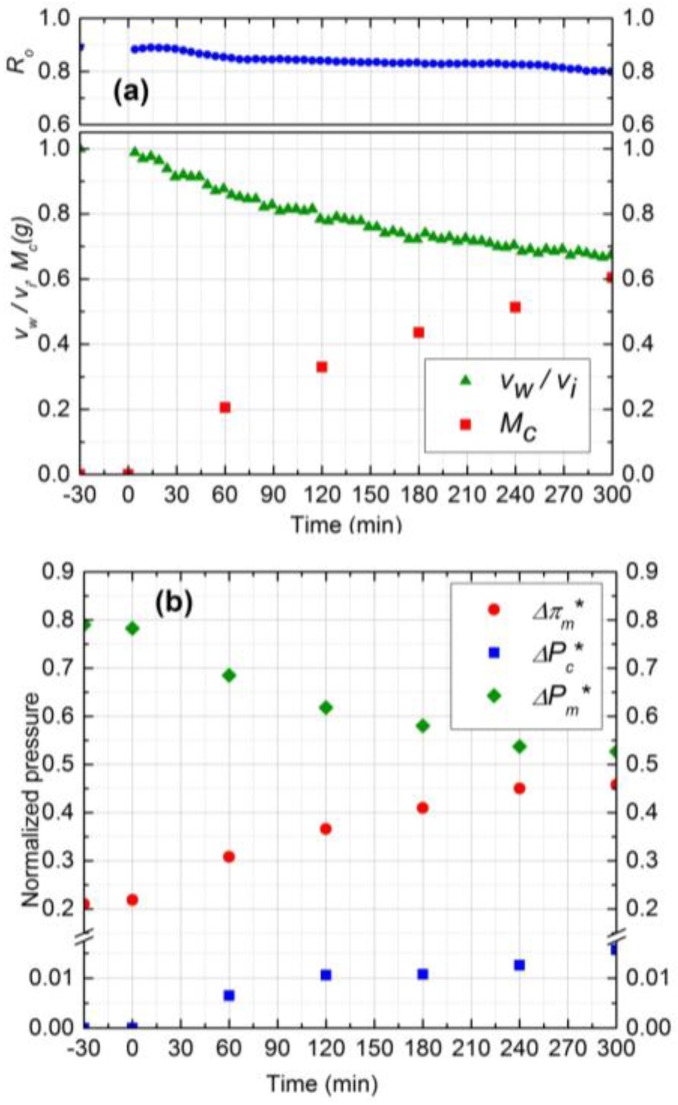
(**a**) Normalized permeate flux (*v*_w_/*v*_i_), Observed rejection (*R*_o_) and Deposited cake mass (*M*_c_) on NF90 membrane. Fouling experiment conducted at 965 kPa, cross-flow velocity 0.1 m/s, 10 mM NaCl solution, 300 ppm of 130 nm Silica, temperature of 25 °C and pH 7.0; (**b**) Comparison of normalized trans-membrane pressure ($\Delta P_{m}^{*}$), trans-cake hydrodynamic pressure ($\Delta P_{c}^{*}$) and CEOP ($\Delta \pi_{m}^{*}$).

**Figure 5:**
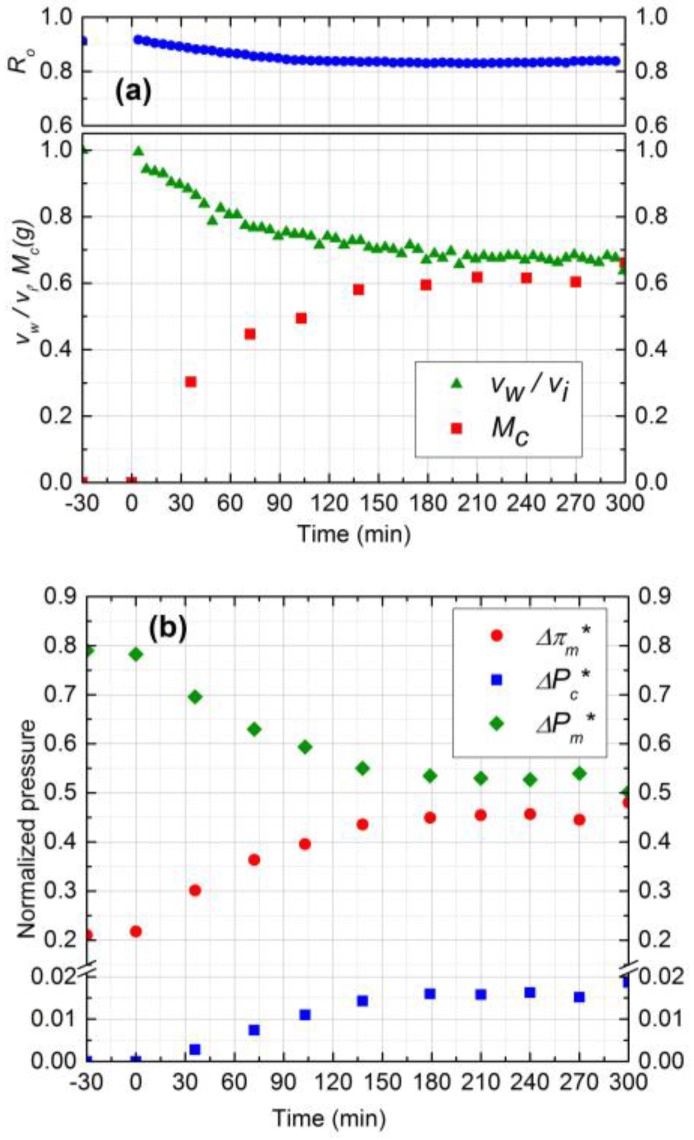
(**a**) Normalized permeate flux (*v*_w_/*v*_i_), Observed rejection (*R*_o_) and Deposited cake mass (*M*_c_) on NF90 membrane. Fouling experiment conducted at 965 kPa, cross-flow velocity 0.1 m/s, 10 mM NaCl solution, 500 ppm of 130 nm Silica, temperature of 25 °C and pH 7.0; (**b**) Comparison of normalized trans-membrane pressure ($\Delta P_{m}^{*}$), trans-cake hydrodynamic pressure ($\Delta P_{c}^{*}$) and CEOP ($\Delta \pi_{m}^{*}$).

Experimental results for two silica concentrations, 300 ppm (run 1) and 500 ppm (run 2), are shown in Figure 4 and Figure 5, respectively. In Figure 4a and Figure 5a, the normalized permeate flux (*v*_w_/*v*_i_), observed rejection (*R*_o_) and deposited mass (*M*_c_) on the membrane are depicted. The initial salt water flux was 1.9 × 10^−5^ m^3^/m^2^s for both experiments. After 5 h of filtration, the permeate flux decreased by 33% and 36% for 300 ppm and 500 ppm, respectively, whereas the observed rejection declined about 8% for both. For 500 ppm silica concentration, the initial deposition and flux decline rate was higher, and the deposition rate decreased significantly after 3 h. The water flux was virtually constant after 3 h owing to the fact that the critical flux for particle deposition was attained in this case and the cake development was arrested. The formation of the cake is primarily governed by the rate of particle deposition on the membrane, which is driven by the permeation drag. As the cake layer thickness increases, this permeation drag decreases, and eventually reaches a critical value, below which no particles can be convected to the cake surface. The critical flux for particle deposition for 500 ppm (~1.20 × 10^−5^ m^3^/m^2^s) was attained after 3 h and remained constant after that. For the 300 ppm concentration, on the other hand, the flux decline continued over the 5 h experiment. Hence, at higher particle concentrations, the mass deposition rate is faster, as evident from the corresponding experimental plots in Figure 4a and Figure 5a.

The cake layer thickness for 300 ppm and 500 ppm silica solutions was calculated to be 37 µm and 41 µm, respectively. As the cake layer thickness is very small (about 1.0% of the hydrodynamic diameter of the channel) the assumption of film theory and constant mass transfer coefficient becomes reasonable [21]. The average porosity values for 300 ppm and 500 ppm obtained by Equations 9 and 10 to be 0.48 and 0.5, respectively.

The relative contribution of CEOP and trans-cake hydrodynamic pressure drop for 300 ppm and 500 ppm are shown in Figure 4b and Figure 5b, respectively. These results were obtained by following the calculation procedure described in Section 2. Normalized trans-cake hydrodynamic pressure was 1.5% of the applied pressure. Due to the growth of the cake layer, the CEOP increased 25% and 28% for 300 ppm and 500 ppm, respectively. Therefore, CEOP is the dominant mechanism for the reduction of permeate flux and observed salt rejection.

#### 3.4.2. Effect of Cross-Flow Velocity

**Figure 6:**
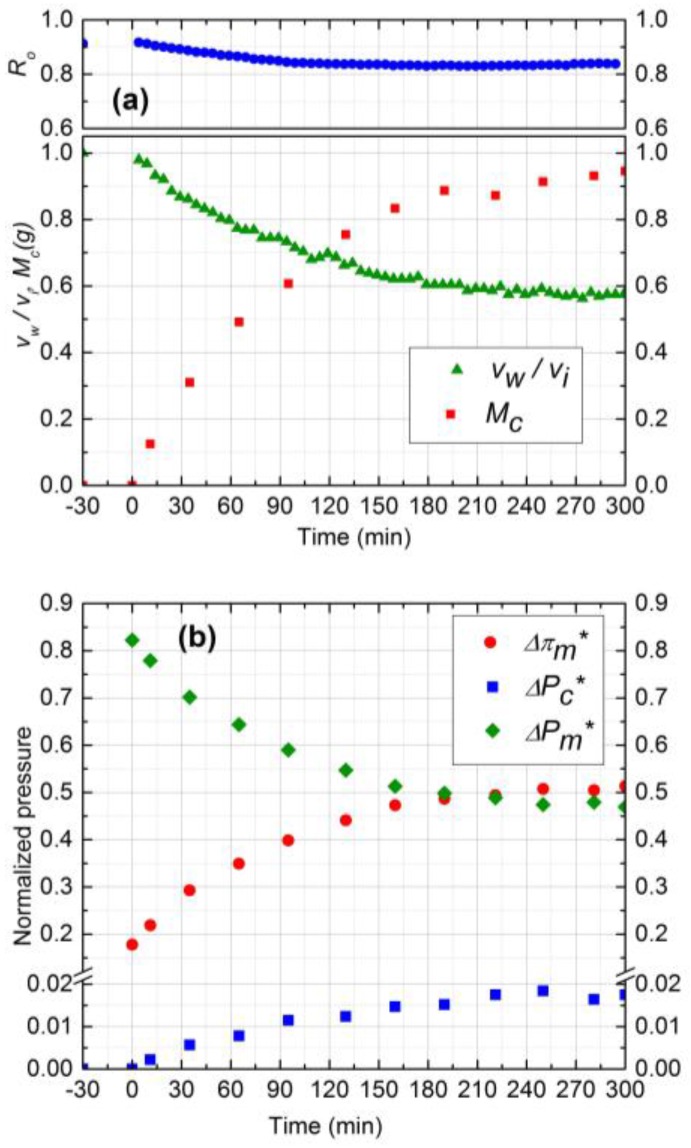
(**a**) Normalized permeate flux (*v*_w_/*v*_i_), Observed rejection (*R*_o_) and Deposited cake mass (*M*_c_) on NF90 membrane. Fouling experiment conducted at 965 kPa, cross-flow velocity 0.2 m/s, 10 mM NaCl solution, 500 ppm of 130 nm Silica, temperature of 25 °C and pH 7.0; (**b**) Comparison of normalized trans-membrane pressure ($\Delta P_{m}^{*}$), trans-cake hydrodynamic pressure ($\Delta P_{c}^{*}$) and CEOP ($\Delta \pi_{m}^{*}$).

The data analysis model outlined in this paper can be used to aid in the interpretation of complex, multi-cause fouling data, as illustrated by the following example. The experimental results for two cross-flow velocities (0.1 and 0.2 m/s) are shown in Figure 5 and Figure 6, respectively (runs 2 and 3). The normalized flux declined by 44% for 0.2 m/s cross-flow velocity as compared to 34% for 0.1 m/s. The observed rejection declined 8% for both experiments. The more severe flux decline for 0.2 m/s (run 3) may at first appear counter-intuitive, because higher cross-flow velocity should have led to lower fouling. This is where the coupling between salt CP and cake filtration manifests itself in an interesting manner. At higher cross-flow velocities, the salt CP and thus the TMOP is lower (Figure 6b), which results in higher initial permeate flux (2.0 × 10^−5^ m^3^/m^2^s). This causes a more rapid initial colloid deposition, resulting in a more aggressive growth of cake. Furthermore, the critical flux is attained at a later time in this experiment compared to experiment 2. Hence, keeping all other parameters constant, increasing the cross-flow velocity increases the initial colloid deposition and aggravates the cake growth. The total mass deposited in experiment 3 after 5 h is higher than experiment 2 (0.95 g compared to 0.65 g). Therefore, the rate of deposition is mostly governed by the permeation drag, and cross-flow velocity seems to have a minor effect on particle removal from cake surface.

The average porosity obtained from experimental mass deposition data was 0.5 and 0.52 for 0.1 m/s and 0.2 m/s, respectively. Since the feed salt concentration (10 mM) and subsequently the electrostatic repulsion of silica particles was similar for both experiments, the cake layer porosity was expected to be similar. The reason for a slight increase in average porosity for 0.2 m/s cross-flow velocity can be attributed to the higher porosity of upper layers of deposited cake as it grows [25]. Cake layer thickness values were calculated to be 41 µm and 55 µm for 0.1 m/s and 0.2 m/s cross-flow velocities, respectively, after the 5 h experiment.

Normalized pressure drops due to CEOP and trans-cake pressure drop are shown in Figure 5b and Figure 6b. The TMOP increased 28% and 35% after 5 h experiment for 0.1 m/s and 0.2 m/s, respectively. The observed increase in CEOP at even higher cross-flow velocity was due to more silica deposition and enhanced hindered diffusivity. For both experiments, the trans-cake pressure drop was very small (less than 2% of applied pressure) compared to CEOP drop. Therefore, enhanced CEOP is the dominant mechanism for the performance decline, which is mostly influenced by the deposited mass or the thickness of the cake layer.

#### 3.4.3. Effect of Operating Pressure

**Figure 7:**
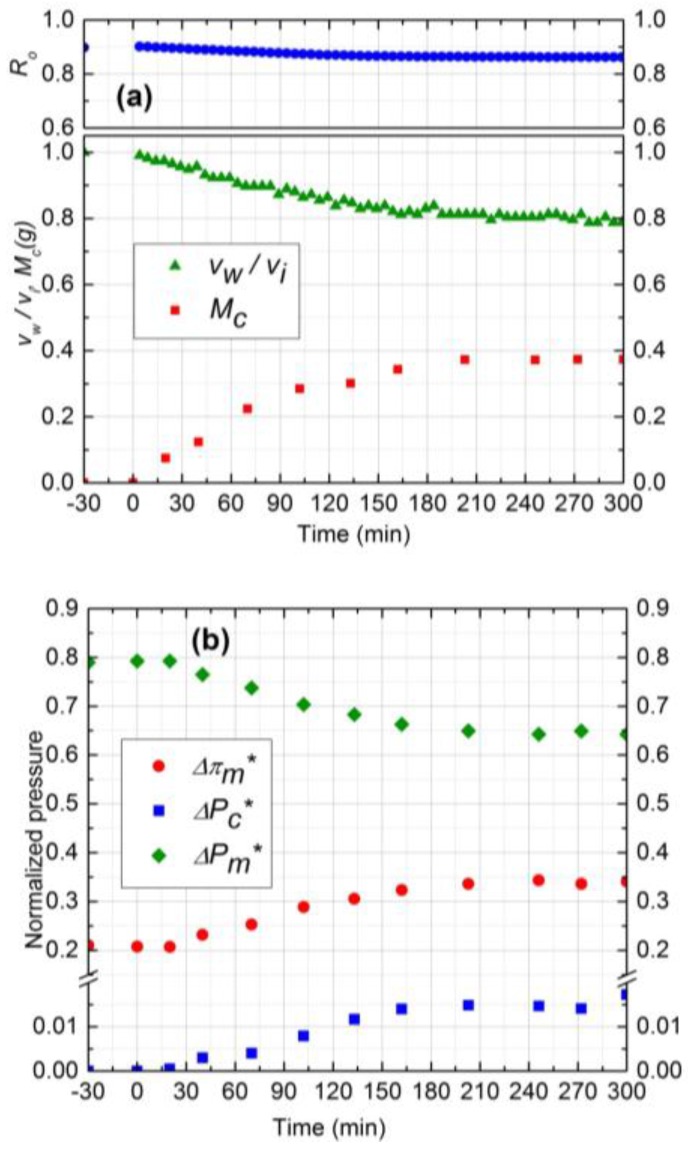
(**a**) Normalized permeate flux (*v*_w_/*v*_i_), Observed rejection (*R*_o_) and Deposited cake mass (*M*_c_) on NF90 membrane. Fouling experiment conducted at 689 kPa, cross-flow velocity 0.1 m/s, 10 mM NaCl solution, 300 ppm of 130 nm Silica, temperature of 25 °C and pH 7.0; (**b**) Comparison of normalized trans-membrane pressure ($\Delta P_{m}^{*}$), trans-cake hydrodynamic pressure ($\Delta P_{c}^{*}$) and CEOP ($\Delta \pi_{m}^{*}$).

To study the effect of operating pressure on initial salt water flux and subsequently on the fouling performance of NF membrane, two experiments (runs 1 and 4) were considered at two different pressures (689 and 965 kPa) with the other conditions remaining identical. Figure 7 shows the results for experiment 4 at 689 kPa operating pressure. According to Figure 4a and Figure 7a, after 5 h, permeate flux and observed rejection decline was higher by 15% and 4%, respectively, for 965 kPa, as compared to 689 kPa. Higher flux decline at 965 kPa is due to the higher and continuous deposition of silica particles during 5 h filtration. On the other hand, the rate of silica deposition reached steady state value after 210 min for 689 kPa as shown in Figure 7a. The steady state mass deposition and permeate flux were 0.37 g and 1.14 × 10^−5^ m^3^/m^2^s, respectively.

The initial salt water flux at 965 kPa was 35% higher compared to 689 kPa and salt concentration was similar. Hence, it was expected that the average porosity of the cake layer at 965 kPa would be lower. However, the average porosity for both experiments was calculated to be 0.48 which is in good agreement with the literature [14]. Based on critical flux values, silica deposition stopped earlier at 689 kPa as compared to 965 kPa and the exerted transmembrane pressure is supposed to make the cake layer denser after that, instead of forming new layers. Hence, in addition to salt water flux and deposited mass, critical flux is also a determining parameter for controlling the average porosity of the cake layer.

Experimental results of normalized pressure drops are shown in Figure 4b and Figure 7b for 965 kPa and 689 kPa, respectively. As the amount of silica deposition was higher for 965 kPa, the CEOP was also higher by 10% compared to 689 kPa. Normalized trans-cake hydrodynamic pressure drop was 1.5% of the operating pressure for both experiments. Therefore, operating at lower pressure is beneficial for the performance of the membrane as it reduces the effect of CEOP considering that the flux will eventually become steady after reaching the critical flux of particle deposition.

#### 3.4.4. Effect of Salt Concentration

**Figure 8:**
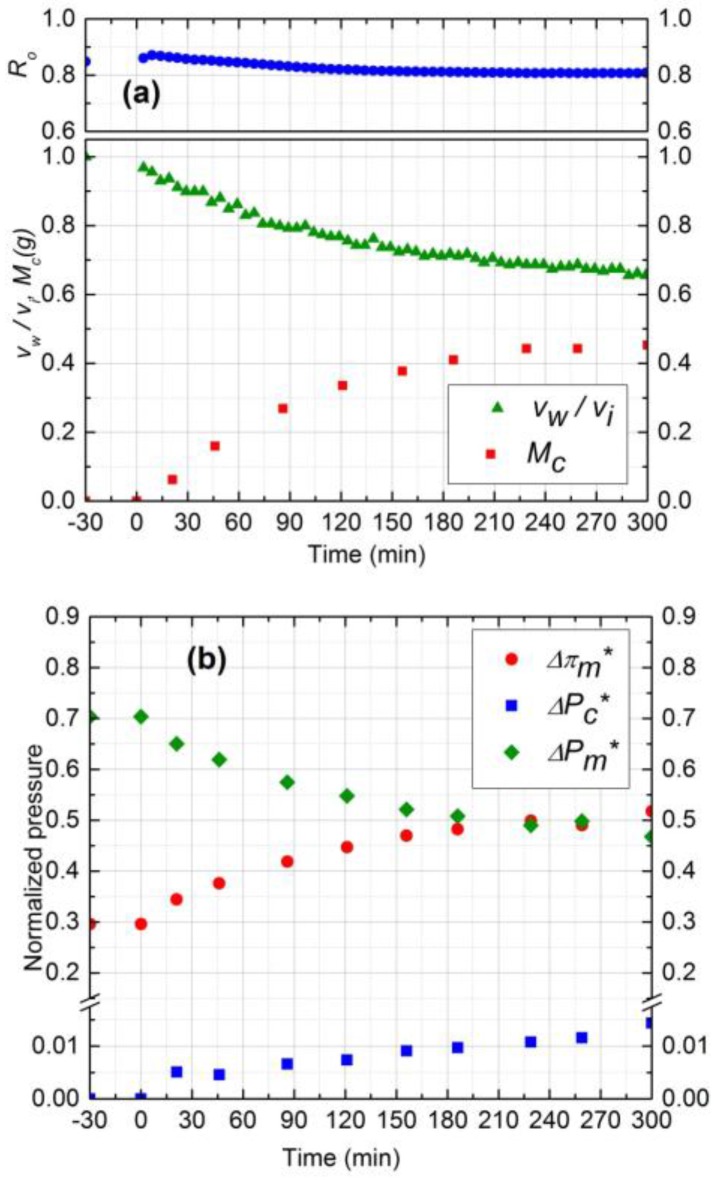
(**a**) Normalized permeate flux (*v*_w_/*v*_i_), Observed rejection (*R*_o_) and Deposited cake mass (*M*_c_) on NF90 membrane. Fouling experiment conducted at 1033 kPa, cross-flow velocity 0.1 m/s, 20 mM NaCl solution, 300 ppm of 130 nm Silica, temperature of 25 °C and pH 7.0; (**b**) Comparison of normalized trans-membrane pressure ($\Delta P_{m}^{*}$), trans-cake hydrodynamic pressure ($\Delta P_{c}^{*}$) and CEOP ($\Delta \pi_{m}^{*}$).

The salt concentration affects the performance of the filtration process by controlling the rate of mass deposition, the porosity of cake, and CEOP. The rate of mass deposition is mainly governed by the initial salt water flux and silica concentration in the feed. As mentioned before, the porosity of the cake layer also depends on initial permeation drag (salt water flux), deposited mass, critical flux, and salt concentration. To study the effect of salt concentration on the cake layer porosity and CEOP, two experiments were considered (runs 1 and 5) at two different salt concentration (10 mM and 20 mM) having the same initial salt water flux. The operating pressure was set at 1033 kPa in run 5 to have the same initial salt water flux as run 1 (Figure 8).

According to Figure 4a and Figure 8a, permeate flux declined 33% and 35% for 10 mM and 20 mM, respectively. The amount of mass deposition after 5 h filtration was 0.15 g less for 20 mM experiment. The average experimental porosity obtained for 20 mM salt concentration was 0.45 which is lower than the porosity of 10 mM experiment, 0.48. The observed higher flux decline, even at low mass deposition, is attributed to the denser structure of cake layer at 20 mM. The decrease of porosity at higher salt concentration is due to the reduced electrostatic repulsion at the same permeation drag condition [22]. CEOP increased 25% for both experiments as shown in Figure 4b and Figure 8b. The similar CEOP, in spite of increase in solute concentration in experiment 5 (Figure 8b), can be justified by looking at the concept of CECP model. According to this model, the osmotic pressure difference highly depends on the porosity and the thickness of the cake layer. The cake layer formed in experiment 1 is thicker while is more porous than that formed in experiment 2. The intensifying effect of higher cake layer thickness on CP in experiment 1 might be counter-balanced by the diminishing effect of lower cake layer porosity in this experiment, and thus the osmotic pressure enhancement was negligible. Based on experiments 1–5, it can be concluded that the critical flux for 130 nm silica particles is around 1.20 ± 0.06 × 10^−5^ m^3^/m^2^s.

Taking a closer at Figure 4, Figure 5, Figure 6, Figure 7 and Figure 8, the significance of CECP model is demonstrated. Based on van’t Hoff equation (*π* = *iCRT*), the osmotic pressure of the 10 mM NaCl solution at 298 K is about 0.44 atm (*i* = 2 theoretically for 1-1 electrolytes like NaCl and *R* = 0.08206 L·atm/mol·K). The CEOP, for example in Figure 4, Figure 5 and Figure 6, is 4.5 ± 2.5 which is almost 10 times higher than the osmotic pressure of the solute in the bulk solution. This observation implies that the NaCl concentration on the membrane surface could be about 10 times higher than that in the bulk which is in agreement with the literature [14]. 

## 4. Materials and Methods

### 4.1. Model Colloids, Membrane, and Reagents

As model colloidal foulant, Snowtex-ZL silica particles (Nissan Chemical America Corporation, Houston, TX, USA) in 40 wt % aqueous suspension having pH 9.0 were used. The specific gravity was 1.12–1.14 and density was 2300 kg/m^3^ [24]. The size and zeta potential of particles were determined using dynamic light scattering (DLS, ALV/CGS-3 Compact Goniometer System, Langen, Hesse, Germany) and DT-1200 Electroacoustic spectrometer (Dispersion Technology, Inc., New York, NY, USA). The average hydrodynamic diameter and zeta potential were 130 nm and −30 mV at pH 7.0 in 10 mM and 20 mM NaCl solution. The NF membrane used for the experiments was an aromatic polyamide composite membrane (NF90, supplied by Dow FilmTec, Edina, MN, USA). The membrane samples were immersed in deionized water and stored at 5 °C. The average roughness of NF90 membrane is 65 nm [26] and zeta potential from streaming potential measurement is −18 mV within the pH range of 7.0–9.0 in 10 mM NaCl solution [27]. The salt solution was prepared by dissolving 99% NaCl (Sigma-Aldrich, Oakville, ON, Canada) in deionized water.

### 4.2. Cross-Flow Membrane Filtration Setup

**Figure 9:**
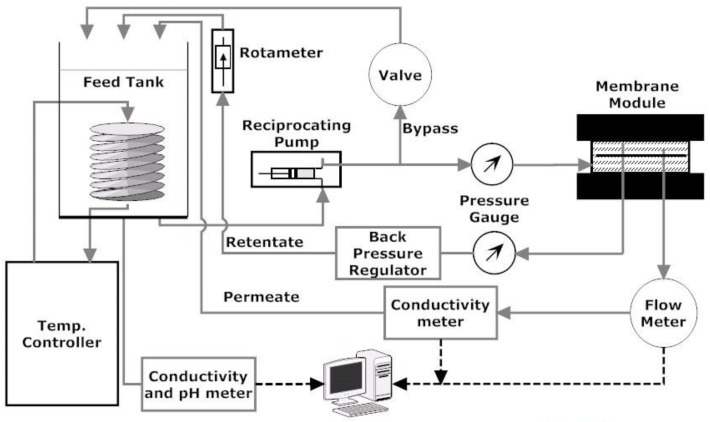
Schematic of cross-flow membrane filtration unit.

The laboratory scale cross-flow membrane filtration setup is shown in Figure 9. The setup is a modified version of commercially available stainless steel Sepa CF cell (Sterlitech Corporation, Kent, OH, USA). The membrane cell has channel dimensions of 14.6 cm × 9.5 cm × 1.7 mm. The effective membrane area and cross-sectional flow area for these dimensions are 1.40 × 10^−2^ m^2^ and 1.62 × 10^−4^ m^2^, respectively. These channel dimensions provide cross-flow velocity values of 0.1 and 0.2 m/s and Reynolds numbers 344 and 688 (laminar) for the experimental condition of 1.0 and 2.0 LPM volumetric cross-flow rates, respectively. A constant flow diaphragm pump of maximum capacity 6.8 LPM (1.8 GPM) from Hydra-Cell was used to provide feed to the Sepa CF cell at a maximum 6895 kPa pressure. The feed suspension was supplied from a 19 L (5 Gal.) stainless steel tank open to atmosphere. A bypass valve was used before the membrane module to adjust feed flow rates. The original setup was modified by replacing the concentrate control valve placed at the channel outlet with a back pressure regulator (Swagelok, Solon, OH, USA). The combination of the bypass valve and back pressure regulator allowed fine and constant control over a wide range of applied pressure and cross-flow velocities within the membrane filtration unit. The applied pressure was monitored using a pressure gauge (Ashcroft, Stratford, CT, USA) installed before the back pressure regulator, and retentate flow rate was monitored using a floating disk rotameter installed after the back pressure regulator. Feed water temperature was maintained at room temperature by a recirculating heater/chiller (Isotemp 3013, Fisher Scientific, Ottawa, ON, Canada). A digital flow meter (Cole-Parmar, Montreal, QA, Canada) of flow range 0 to 100 mL/min and two conductivity probes (Fisher Scientific, Ottawa, ON, Canada) were used to measure the permeate volume, feed, and permeate conductivity, respectively. The results were collected directly into a computer using the data acquisition system developed using LabVIEW (National Instruments, Austin, TX, USA).

### 4.3. Experimental Protocol

Before each experiment, the membrane was compacted for 2 h at 1515 kPa. After that, the applied pressure and cross-flow velocity were set to the desired condition of the filtration experiment. The pure water flux ($v_{w}^{0}$) was measured for 1 h and membrane resistance (*R*_m_) was calculated for each membrane. Then, NaCl solution was added to obtain the desired feed salt concentration (*C*_i,f_). The salt water flux ($v_{w}^{s}$) and observed rejection (*R*_o_) were measured during 1 h of equilibration. The permeate and retentate were returned to the feed tank to maintain a constant salt concentration in the feed. The pH was maintained at 6.8 ± 0.2 for all the experiments. After electrolyte equilibration, colloidal silica particles were added to the feed tank to provide an appropriate colloid concentration in the feed (*C*_p,f_). The operating pressure and cross-flow velocity were maintained at the same values as for the electrolyte equilibration step. The permeate flux (*v*_w_) and observed rejection (*R*_o_) were measured over a 5 h fouling experiment. The pH of the feed solution was also measured at the beginning and end of each fouling experiment. The silica concentration in the feed was measured by UV absorbance (UV-VIS Spectrometer, Varian Cary® 50, Agilent Technologies, Santa Clara, CA, USA). The path length for the quartz UV absorbance cell was 10 mm and a wavelength of 225 nm was used to minimize the effect of NaCl absorbance.

### 4.4. Characterization of Silica Particles

It is important to characterize the colloidal particles to mechanistically explain the fouling experimental results. Therefore, the particle size and zeta potential were measured before the fouling experiments. To measure the size and zeta potential of the silica particles, a solution of 10 mM NaCl in deionized water was prepared. Next, 3.125 g of the Snowtex ZL dispersion with pH 9.0 was weighed in a beaker. The colloidal dispersion was diluted to 125 g total weight using 10 mM NaCl solution to prepare 1.0 wt % colloidal suspension in 10 mM NaCl solution. HCl or NaOH was added to adjust the pH of the samples. After that, the samples were stirred for 12 h and sonicated for 1 h to make sure that silica particles are not aggregated. Finally, the size of the silica particles was measured using DLS and DT-1200 Acoustic spectrometer. The zeta potential of the Snowtex ZL particles were also measured at different pH for ionic strength of 10 mM NaCl and temperature of 25 °C using an Electroacoustic spectrometer.

### 4.5. Membrane Compaction

Before conducting colloidal fouling experiments, it is necessary to compact the membranes hydrostatically to acquire constant membrane properties in terms of water permeation. Different water flux values obtained by a new membrane (before compaction) and a used one (after compaction) demonstrate why membrane pre-compression is necessary. Membrane compaction experiment was conducted by increasing and decreasing the operating pressure stepwise from 275 to 1240 kPa and 1240 to 275 kPa, respectively, for the new membrane. The permeate flux was monitored for 15 minutes at each pressure. Then, the same experiment was conducted after compacting the membrane at 1515 kPa for 2 h. In both experiments, the cross-flow rate was adjusted at 1 LPM. To determine the compaction effect, the permeate flux or the membrane residence are typically plotted as a function of time or pressure and the presence of hysteresis is studied.

### 4.6. Critical Flux Measurement

**Figure 10:**
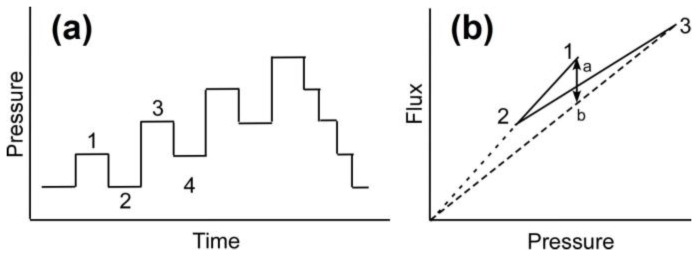
(**a**) Pressure vs. time in pressure step method and (**b**) corresponding flux vs. pressure. The flux of step 4 is included on segment a–b [28] (Copyright 2002, Reproduced with permission from Elsevier Science Ltd., Oxford, UK).

The filtration experiments must be conducted at fluxes higher than critical flux to ensure that colloidal fouling is happening. The experimental protocol to determine the critical flux is based on the pressure step method [28]. In this method, each steady state flux measurement at an applied pressure is followed by a decrease in applied pressure to determine the reversibility or irreversibility according to Figure 10a. The advantage of this method is that it allows a rigorous determination of the critical flux above which irreversible colloidal fouling occurs.

By comparing the corresponding flux obtained at pressure steps 1 and 4 in Figure 10b, one can determine whether the flux achieved in step 3 is due to irreversible (cake formation) or reversible (CP layer) fouling phenomenon. According to Figure 10b if the flux in step 4 is on point b, fouling is irreversible, or cake formation occurs at the membrane surface, and if the flux is on point a, fouling phenomenon is by CP. Therefore, reversible or irreversible fouling can be determined according to the flux value at step 4 (included on segment a–b).

The procedure to estimate the critical flux in this study included (1) compaction for 2 h at 1515 kPa; (2) preparation of 10 L solution with 10 mM NaCl and 300 ppm of 130 nm silica; (3) applying pressure in sequence order: 275–345–275–415–345–...–750 kPa (Figure 10a); (4) reducing the applied pressure stepwise from maximum (750 kPa) to minimum (275 kPa) pressures. At each pressure, flux was monitored for 20 min to ensure stable performance. The pressure was increased until critical flux or irreversibility in flux measurement was obtained.

### 4.7. Calculation of the Deposited Mass

The mass of colloidal cake layer was determined by measuring feed solution concentration at a different time interval and conducting simple mass balance of the feed suspension. Considering the initial (*t* = 0) feed concentration is *C*_p,0_ and volume is *V*_f,0_, the total mass at this time will be *m*_0_ = *C*_p,0_*V*_f,0_. At *t* = *t*, the feed concentration is *C*_p,*t*_ and sample volume remains constant (*V*_f,0_) since both permeate and retentate are recycling back to the feed tank (Figure 9). Therefore, the mass of silica in feed at time *t* is *m_t_* = *C*_p,*t*_*V*_f,0_. The feed concentrations (*C*_p,*t*_) at different times were measured by UV absorbance analysis using a UV-VIS Spectrometer (Varian Carey 50). The path length for the UV-absorbance experiment was 10 mm and wavelength was chosen 225 nm to minimize the effect of NaCl solution absorbance. During the UV absorbance analysis, scan mode of the instrument was used instead of simple read. This method allowed UV absorbance measurement of the sample over a wide range of wavelength and provided more flexibility to use specific wavelength for calculation. The mass reduction from the tank during time *t* was the amount of mass deposited on the membrane surface and was calculated by subtracting *m_t_* from *m*_0_ (*M*_c_ = *m*_0_ − *m_t_* = *C*_p,0_*V*_f,0_ − *C*_p,*t*_*V*_f,0_). The calculated *M*_c_ is then used in Equation (5) to find the hydraulic resistance of the cake layer.

## 5. Conclusions

A standard experimental and data analysis methodology was developed to scientifically conduct colloidal fouling experiments using NF membranes. The significant effect of membrane compaction on water flux and the observed hysteresis clearly showed the dynamic behavior of the membranes with the trans-membrane pressure. Pre-conditioning of the NF membranes at higher pressures than the fouling experiment pressure was found to be essential for proper correlation of the flux/rejection behavior with the colloidal fouling. In order to determine the effect of colloidal particle and salt concentrations, applied pressure and the cross-flow velocity on the permeation properties of the membrane, critical flux, deposited mass, porosity of cake layer, trans-cake and trans-membrane hydraulic pressures, and CEOP were measured. A significant contribution of this study is to provide a detailed description of the applied methodologies for measurement of these parameters. It was found that the flux/rejection behavior was governed by the synergistic effects of the initial flux, critical flux, the density of the cake layer, deposited mass, and CEOP. The coupling between salt CP and colloidal fouling was seen to manifest in a complex manner that depended on salt concentration, as well as colloidal particles and membrane characteristics (e.g., surface charge). The data analysis model developed presented herein was successfully used to explain the counter-intuitive experimental results. This study provides valuable guidelines for researchers who are working on experimental and theoretical aspects of combined colloidal fouling and salt CP using salt rejecting membranes.

## Figures and Tables

**Table 1:** Experimental conditions for fouling experiment on NF90 membrane at 25 ± 1 °C and pH = 7.0.

Exp. No.	Operating Parameters			
	Δ*P* (kPa)	*C*_p,f_ (ppm)	*C*_i,f_ (mM)	*u* (m/s)
1	965	300	10	0.1
2	965	500	10	0.1
3	965	500	10	0.2
4	689	300	10	0.1
5	1033	300	20	0.1

**Figure A1:**
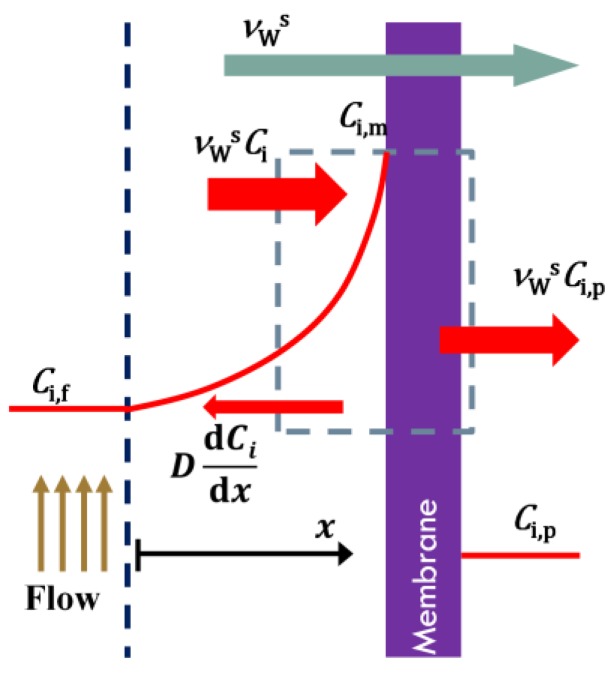
Schematic diagram of the CP layer.

## Appendix A

Equation 4 is a basic equation in the mass transport through the membranes which is derived using van’t Hoff equation and film theory. Schematic diagram of the CP layer formed on the membrane is shown in Figure A1. Writing the mass balance for the salt on a control volume shown in this figure gives the following equation [17,29,30]:

(A1) $$\nu_{W}^{s}C_{i}-\nu_{W}^{s}C_{i,p}-D\frac{dC_{i}}{dx}=0$$

The boundary condition is as follows:

(A2) $$\begin{array}{l} x=0\;C_{i}=C_{i,f}\\ x=\delta \;C_{i}=C_{i,m} \end{array}$$

where *δ* is the thickness of the mass boundary layer. Applying these boundary conditions, the boundary layer film model is derived:

(A3) $$\frac{C_{i,m}-C_{i,p}}{C_{i,f}-C_{i,p}}=\exp\left(\frac{\nu_{W}^{s}\delta }{D_{i}}\right)=\exp\left(\frac{\nu_{W}^{s}}{k_{i}}\right)$$

where *D*_i_ and *k*_i_ are the diffusion and mass transfer coefficients of salt in water, respectively, and *C*_i,m_ is the salt concentration at the membrane surface. The van’t Hoff equation gives the osmotic pressure difference for the simple case of dilute 1-1 electrolyte as following:

(A4) $$\Delta \pi =2RT\left(C_{i,m}-C_{i,p}\right)$$

Plugging in $C_{i,m}-C_{i,p}$ from Equation (A3) in Equation (A4) gives:

(A5) $$\Delta \pi =2RT\left(C_{i,f}-C_{i,p}\right)\exp\left(\frac{\nu_{W}^{s}}{k_{i}}\right)$$

Equation (4) is finally derived replacing $C_{i,f}-C_{i,p}$ using the salt rejection equation (Equation (2)).
